# Implementation of a worksite educational program focused on promoting healthy eating habits

**DOI:** 10.12688/f1000research.2-201.v2

**Published:** 2014-03-07

**Authors:** Dimitra Tanagra, Dimitris Panidis, Yannis Tountas, Elina Remoudaki, Evangelos C. Alexopoulos

**Affiliations:** 1 Postgraduate Course of Health Promotion & Education, Medical School, University of Athens, Athens, Greece; 2 Laboratory of Biopharmaceutics and Pharmacokinetics, School of Pharmacy, University of Athens, Athens, Greece; 3 Department of Occupational Health, Onassis Cardiac Surgery Center, Athens, Greece

## Abstract

**Objective: **To estimate the effectiveness of a short-term educational-counseling worksite program focused on lipid intake, by monitoring the possible change on nutrition knowledge and eating habits.

**Methods: **an 8-week educational program based on the Health Belief Model was implemented in a honey packaging and sales company in Greece. 20 out of the 29 employees initially enrolled completed the program. Knowledge level and eating habits were evaluated prior and after the intervention by the “Nutrition Knowledge Questionnaire” and the “Food Habits Questionnaire”. ANOVA, Spearman rho test and paired Wilcoxon test were employed in statistical analysis.

**Results: **Non smokers and those with higher educational level had healthier eating habits. Knowledge following the intervention was significantly improved concerning recommendations and basic food ingredients but as far as eating habits were concerned, scores were not improved significantly, while intake of fried food was increased.

**Conclusions and Implications**: Short-term interventions may produce substantial improvement in knowledge but not necessarily modifications in unhealthy eating habits.

## Introduction

Over the last decades, obesity has rapidly turned into a global epidemic in both developed and developing countries, affecting adults, children and adolescents as well. Currently, the number of people suffering from obesity is estimated at approximately 400 million people worldwide ^1^. Moreover, increased body mass index (BMI) is associated with higher risk of cardiovascular diseases, some types of cancer and type II diabetes ^2– 4^. Recent data from Greece have shown obesity is an epidemic problem ^5, 6^. In recent years, Greeks have abandoned the traditional Mediterranean diet; one study reports that only 33% of Greek men and 43% of Greek women adhere to a traditional Mediterranean diet ^7^.

Among various individual and lifestyle factors, many work-related factors are responsible for the modification of dietary patterns including working conditions, such as: working overtime, high job demands, occupational stress and others ^8^. On the other hand, the workplace has been identified as a promising setting for health promotion although the findings of many worksite health promotion (WHP) programs indicate that these programs are associated with only moderate improvement in dietary intake ^9^. Furthermore, it was shown that diet mediterranisation is feasible in a food-at-work intervention, affecting lunch consumption at the workers canteen ^10^. However in a systematic review, participation levels in health promotion interventions at the workplace were typically below 50% ^11^.

The purpose of the present study was to evaluate the effectiveness of an educational worksite intervention focused on lowering fat intake, by affecting nutrition knowledge and eating habits.

## Methods

### Study population

The 48 employees working in the factory premises of a honey company, were asked to participate in this study. Most of the 48 employees were employed in jobs that required mild to moderate manual and intellectual activity (blue collar workers) and five were employed as food scientists and technologists, and supervisors. No inclusion or exclusion criteria were used and as there were no medical contraindications for participation in the program, as judged by the occupational health physician, all employees were eligible for participation. Twenty-nine employees responded positively (60%) to the invitation and gave their written informed consent. The Medical School Review Board judged that further approval was not required, since this program was under the occupational physician’s supervision and control. During the program, seven employees failed to attend day 2 and/or 3 and another two did not return the final questionnaires and all nine were excluded from the final analysis (see Table 1).

**Table 1.** Description of the health promotion program.

Phase	Description	Duration	Participants
0	Informing workers about the health promotion program	0.5 h	48
1: day 1	Presentation and distribution of the questionnaires Measurements of blood pressure/weight/height	1.5 h	29
2: day 16	Lecture, discussion, distribution of printed material	2 h	24
3: day 23	Issues derived from questionnaire analysis (knowledge gaps) Restrictive factors and alternative suggestions (bad habits)	2 h	20
4: day 45	Redistribution of the questionnaires	0.5 h	20
5: day 52	Final meeting, results presentation, individual counseling	3 h	20

### Survey questionnaires

Initially, all employees were informed about the program and were asked to participate by signing informed consent. Two questionnaires were used in order to estimate (Q1) the employees’ nutrition knowledge level and (Q2) their eating habits (see Supplementary File Q1 and Supplementary File Q2). The questionnaires were translated into Greek by two bilingual expert nutritionists and were piloted in 10 college students and blue collar employees for linguistic validation. Both questionnaires in the pre- and post- intervention phase were self-administered. In order to avoid any confusion or misunderstanding, especially in the “Diet Habits” questionnaire (a part of which is proposed to be administered by an interviewer), we had previously explained the way of answering the questionnaire by the means of an oral presentation and we had also reformatted that part of the “Diet Habits” questionnaire so that the sequence of questions/answers was clear.

Nutrition knowledge was assessed using the “ *Nutrition Knowledge Questionnaire”* (see Supplementary File Q1) ^12^. The questionnaire covers four sections: (i) knowledge on experts' recommendations regarding the optimum intake of different food groups (maximum score: 11); (ii) nutrient knowledge, (maximum score: 69); (iii) food choice (which asks people to choose between different options, e.g. to pick the snack that is low in fat and high in fibers), (maximum score: 10); and (iv) the relationships between diet and disease (maximum score: 20). This last section looks at beliefs about the associations between food type, food quantity and diseases.

The eating habits of the participants were assessed by the “ *Food Habits Questionnaire”* (see Supplementary File Q2) ^13, 14^, which has been widely used to estimate dietary changes ^15, 16^. Questions were rated on a 4-point scale, where 1 reflects the healthiest and 4 the unhealthiest eating habits, respectively. The questionnaire included five sections regarding the following habits: (i) replacing high fat foods with low fat substitutes (score range: 7–28); (ii) modifying high fat foods, e.g. fat removal from meat (score range: 3–12); (iii) avoiding high fat cooking methods (fried food) (score range: 4–16); (iv) consumption of fresh fruit and vegetables as a snack (score range: 3–12); and (v) choosing specially manufactured low fat foods products instead of high fat ones (score range: 5–20). The total score of eating habits is calculated from the sum of section scores divided by 5 (ranged from 4.4 to 17).

Data on age, family status, children, educational level, job position, smoking habit, BMI, arterial blood pressure and number of cigarettes/years of smoking were also collected (see Table 2). The participants’ population reflects sufficiently the general healthy Greek population given that almost 40% of them were overweight or obese and 45% were smokers ^17^.

**Table 2.** Demographic and individual characteristics of the intervention group (n=20).

**Demographic characteristics**	
Age in years *(mean ± SD)*	44.6 ± 9.1
≤ 35 *(n (%))*	4 (20)
36–44	6 (30)
≥ 45	10 (50)
Female *(n (%))*	18 (90)
Family status *(n (%))*	
Married, living with other people	18 (90)
Divorced, living alone	2 (10)
Parenthood *(n (%))*	19 (95)
Educational level *(n (%))*	
< 6 years (elementary)	7 (35)
6–9 years (basic)	4 (20)
9–12 years (high or technical school)	7 (35)
> 12 years	2 (10)
**Individual characteristics**	
ΒΜΙ kg/m ^2^ *(mean ± SD)*	26.5 ± 6.2
< 25 kg/m ^2^ *(n, %)*	11 (61)
25–29.9 kg/m ^2^ *(n,%)*	4 (22)
> 30 kg/m ^2^ *(n, %)*	3 (17)
Smoking	
Smoker *(n, %)*	9 (45)
Pack-years *(mean ± SD)*	8.9 ± 11.6
Non smokers *(n, %)*	11 (55)

### The intervention

The intervention took place in three distinct phases over a total of 7–8 weeks ( Table 1) and it was based on the Health Belief Model which suggests that health behaviors are determined by health beliefs and readiness to take action. Behavioral theory has increasingly been used to guide nutrition research to improve intervention efficacy. The Health Belief Model was developed in the 1950s to explain health behavior associated with the failure of people to participate in programs that would reduce disease risk. Constructs central to the HBM consist of perceived susceptibility, perceived severity, perceived benefits, perceived barriers, and other mediating variables. The construct of self-efficacy is frequently included in applications of the HBM ^18, 19^. The choice of the HBM was based both on the specific environmental context and on previous requests of employees on ways towards healthy eating choices and habits, both at work and home. Their interest was mostly healthy choices and on their relevant barriers and less on obesity or disease related risk perception. The environmental context, which is considered equally important in worksite health promotion interventions, was not addressed in our study since almost all employees, during the paid 30 minute meal break, were using homemade food or snacks. A well-equipped and sufficiently large eating room and kitchen was available, so that employees could heat, store safely and consume their own food. Consequently, we have tried to combine the educational measures with suggestions on strategies for change i.e. restrictive factors and bad habits and other issues derived from questionnaire analysis (see below, program phase 3).

In our program (Phase 1), all employees who initially responded to the invitation attended a 30 minute meeting in which a brief presentation of the self-administered questionnaires was done and instructions about the proper completion of both questionnaires were given. Further clarifications were answered the following days during the collection, where necessary. Data on individual characteristics (age, marital status, children, education, smoking status etc.), were also collected and blood pressure, weight and height of the subjects were measured (Seca® 764, Sigma Medical Co, Athens, Greece) at the end of the meeting. Completed questionnaires were collected, recorded in an electronic database and statistically analyzed. In Phase 2, 15 days after the questionnaires were initially distributed, a 45-minute lecture on healthy eating and mediterranean diet was held followed by discussion and distribution of printed material with practical proposals for adoption of healthier eating habits. Overall, the whole session lasted approximately two hours. A week later a second meeting took place (Phase 3) in order to discuss and clarify issues derived from the conclusions of the initial analysis. Specifically, knowledge gaps and restrictive factors for the adoption of healthier nutritional choices were further discussed. The intervention was completed 22 days later (Phase 4) when the participants were asked to fill in the questionnaires again. In the last phase (5 ^th^), a final meeting took place to present and discuss the results, and for individual counseling by the research team.

### Statistical analysis

Analysis of variance (ANOVA) was used to reveal statistically significant differences among various subgroups. Due to the small sample, Spearman rho test was used to examine correlations of the quantitative variables while the paired signed Wilcoxon test was used to compare average values of continuous variables for each category of nominal variables in the intervention group (before and after). A p-value of <0.05 was considered statistically significant. Statistical processing and data analysis were performed using commercial software (SPSS version 16.0, SPSS Inc., 2007).

## Results

### General characteristics of the study population

From the 29 workers who initially responded positively, 20 workers (67%) attended all phases and completed the WHP program ( Table 1). Losses were mainly due to absences on the days of intervention, or inability or failure to return the study questionnaires in time. Between the final and initial groups there were no significant differences in any of the variables.

Analysis of the knowledge questionnaire, returned by the 29 workers who initially responded, showed a lack of knowledge of food composition in saturated fat, fibers, and salt, of the origin of fatty acids (monosaturated, polysaturated and saturated) and of the sources of antioxidant vitamins. Education level correlated significantly with the partial score, i.e. the higher the education level the higher the scores.

Analysis of the habits questionnaire showed that dietary habits included medium to large consumption of fatty foods. However, the score of the workers corresponding to the avoidance of fried foods was pretty high, reaching almost the excellent level. No significant correlations were found between variables under study and the two first subscales (replacement of fatty foods and meat modification). Men, people living with others, and those without children had a tendency to avoid the more fatty substances. Meanwhile, women consumed less fried foods.

Post-intervention analysis was done in the 20 workers who had participated in all phases. Table 2 shows the demographic and individual characteristics of these workers. Women and blue collar workers accounted for 90% and 95%, respectively, a fact that limits the possibility of revealing significant effects of these variables (sex and job title) on the results of the intervention.

As expected, there were significant correlations between the sections (scales) of the two questionnaires prior to, and following, the intervention. By contrast, between the different questionnaires, scales were less and weakly correlated with the exemption of the group of the avoidance of fatty substances. Very high Cronbach alpha score (above 0,80) shows satisfactory reliability of all subscales of both questionnaires. Table 3 presents the scores per category (section) of nutritional knowledge prior to, and following, the intervention in the 20 workers. Significant improvement was seen in the sections of “ *dietary recommendations*”, in “ *basic food ingredients*” and in the total score. Prior to intervention, non-smokers had higher (better) scores concerning the subscales of the “ *basic food ingredients*” (41.0 vs 33.1, p=0.08) and the “ *selection of healthier foods*” (6.2 vs 4.8, p=0.06) but these differences did not reach statistical significance. Following the intervention, non-smokers improved more in the “ *selection of healthier foods*” (6.8 vs 5.2, p=0.04).

**Table 3.** Score comparison of population distribution per knowledge category prior to and following the intervention (n=20).

Knowledge category	Max score	Prior to the intervention			Following the intervention			Paired difference		
		Median	Mean	SD	Median	Mean	SD	Mean	95% CI	p-value
Dietary recommendations	11	7	7.13	1.81	8	8.00	1.76	-0.88	-1.68 -0.07	0.035
Basic food ingredients	69	39	37.45	9.99	48	44.05	12.01	-6.60	-11.85 -1.35	0.016
Selection of healthier foods	10	6	5.55	1.70	6	6.10	1.74	-0.55	-1.33 0.23	0.157
Diet – health relationship	20	14	12.50	4.80	15	13.20	4.456	-0.70	-2.92 1.52	0.518
**Total score**	110	66	62.63	15.40	77	71.35	16.39	-8.73	-15.55 -1.90	0.015

Table 4 presents the dietary habit scores prior to, and following, the intervention. The mean score was improved in the categories of “ *replacement of fatty foods*”, “ *meat modification*”, “ *consumption of food and vegetables*”, “ *avoidance of fatty substances*” and in the total score but the difference was far from significant. On the contrary in the habit of “ *avoidance of fried foods*”, the score was significantly worse, a paradox that might be explained by the very high initial score (tendency towards regression to the mean).

**Table 4.** Comparison of dietary habits score per dietary habit category prior to and following the intervention (n=20).

Dietary habits	Max score	Prior to the intervention			Following the intervention			Paired difference		
		Median	Mean	SD	Median	Mean	SD	Mean	95% CI	p-value
Replacement of fatty foods	28	21	20.11	7.35	18	19.15	6.77	0.97	-2.45 4.39	0.560
Meat modification	12	5.50	6.88	3.85	6	6.60	2.41	0.28	-1.14 1.69	0.690
Avoidance of fried foods	16	6	6.12	1.44	7	7.03	1.30	-0.92	-1.56 -0.27	0.008
Food and vegetables consumption	12	8.50	7.92	2.33	8.50	7.67	2.47	0.25	-0.61 1.11	0.549
Avoidance of fatty substances	20	13	13.54	2.95	13	13.08	4.20	0.46	-1.39 2.31	0.610
**Total score**	88	11.44	10.84	2.31	11.79	10.45	2.60	0.39	-0.62 1.40	0.470

A lower score on this assessment indicates improved/more healthy eating habits.

Workers with a normal BMI exhibited better habits compared with overweight and obese subjects in terms of “ *meat modification*” (4.6 vs 12.7, p=0.002). Non-smokers had lower scores compared with smokers in “ *fat avoidance*” (12.6 vs 15.0, p=0.06) and in “ *avoidance of fried food*” (6.5 vs 7.7, p=0.05).

## Discussion

In the present study, a short-term intervention regarding eating knowledge and habits was implemented in a worksite. Knowledge was significantly improved following this intervention, while no significant improvement was achieved concerning dietary habits. The paradox regarding the fact that average consumption of fried, browned or breaded food (“avoidance of fried food”) increased following the intervention could be partially attributed to the very high (excellent) initial score combined with the fact that some national holidays (where meat consumption is imposed by Greek orthodox religion) also coincided with the program.

The finding that knowledge gain did not lead to habit modification may be explained by the short duration of the program and the complexity that characterizes the conscious or unconscious choices of adults. Health promotion programs in workplaces have shown to be cost-effective, especially for long-term interventions ^20, 21^. As already discussed emphasis was given in promoting healthy eating choices and habits as well as in overcoming the relevant barriers. The fact that the Diet-Health relationship, showed the least significant change following the intervention may be attributed to the minimal interest of employees to control health risks. In our study, a number of factors, i.e. education level, job title, family situation and smoking status was shown to be related to the level of nutrition knowledge and dietary habits. However, the small number of participants prevented these correlations to be concurrently analyzed in multivariate analysis. Other factors known to influence dietary behavior including socio-economic factors, stress and organizational factors (increased work demands, low skills motivation, overtime employment) were not analyzed in our study ^22, 23^ but in our setting, the population was highly homogeneous and most of these factors are not anticipated to have a significant discriminatory impact. However, recent findings show that these interventions can be easily incorporated into the daily working routine programs, and if combined with stress management programs, may result in better outcomes ^24, 25^.

Limitations of this study arise from the small sample size and the short duration of the program. The good general health (healthy worker effect) of participants might also have diminished their scope for demonstrating improvements while self-reporting bias seemed to have negligible effect. It would be recommended in future research in similar settings to use additional back-up measures, such as 24 hour recall diaries, in order to enhance the validity of the data. For organizational reasons, we did not attempt to allocate a control group by randomization. On the other hand, the homogeneity of the population concerning socio-economic aspects, the supportive environment, and the good relationships between colleagues are considered to minimize confounding of the different factors.

Short-term interventions may produce substantial improvement in knowledge but not necessarily accompanied by changes in unhealthy eating habits. These types of programs are not far from those commonly encountered in every day practice but do not seem to be effective in changing unhealthy eating habits. Participation by the employees in defining their needs and priorities; planning long-term interventions, and incorporating self-empowerment and stress management techniques might be necessary for cost-effective worksite health promotion programs to succeed in reducing unhealthy eating habits.

## Figures and Tables

**Supplementary File Q1:**
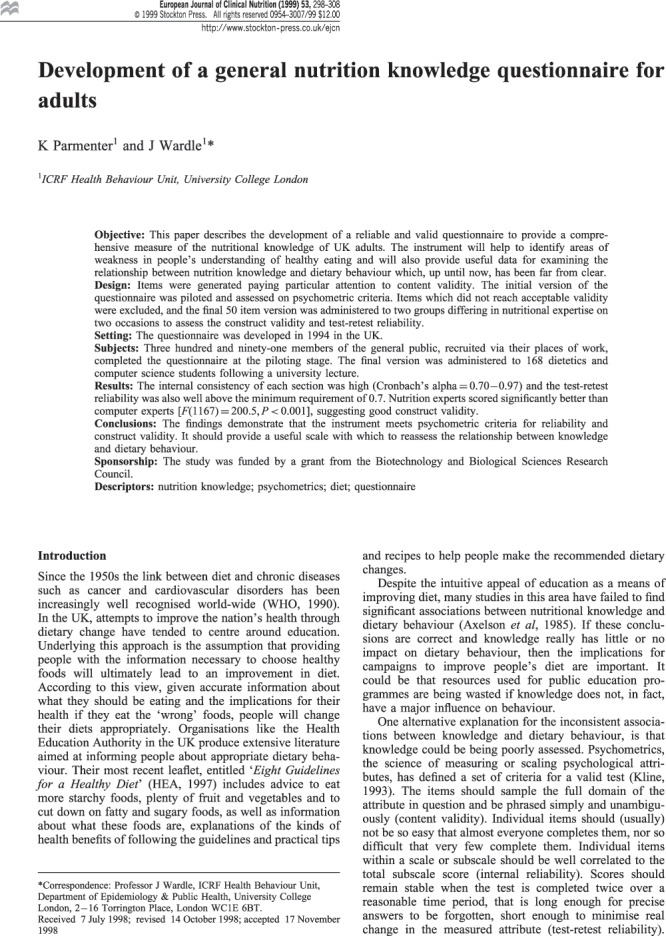
(Sheet 1).

**Supplementary File Q1:**
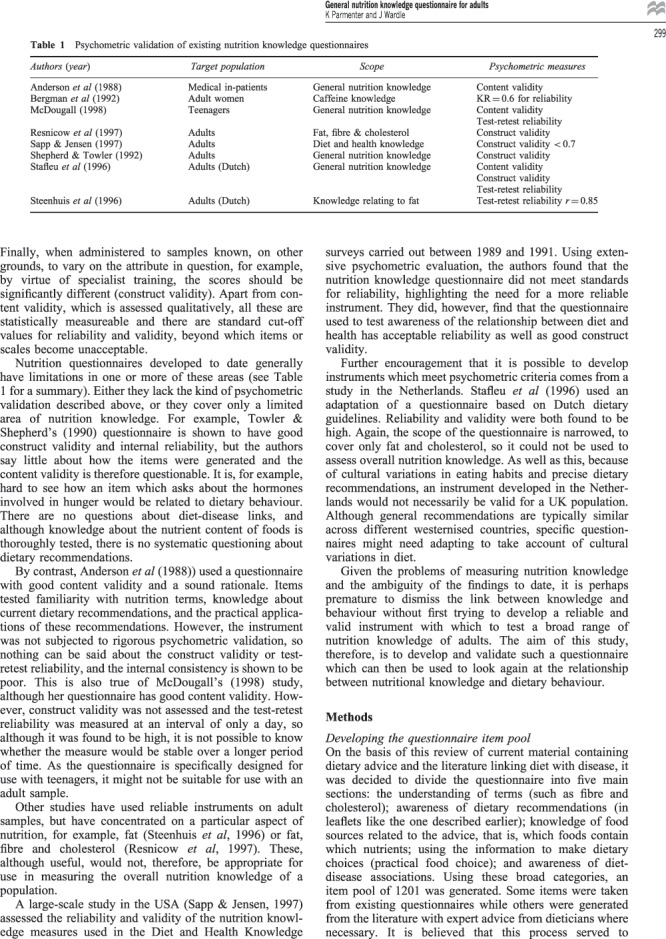
(Sheet 2).

**Supplementary File Q1:**
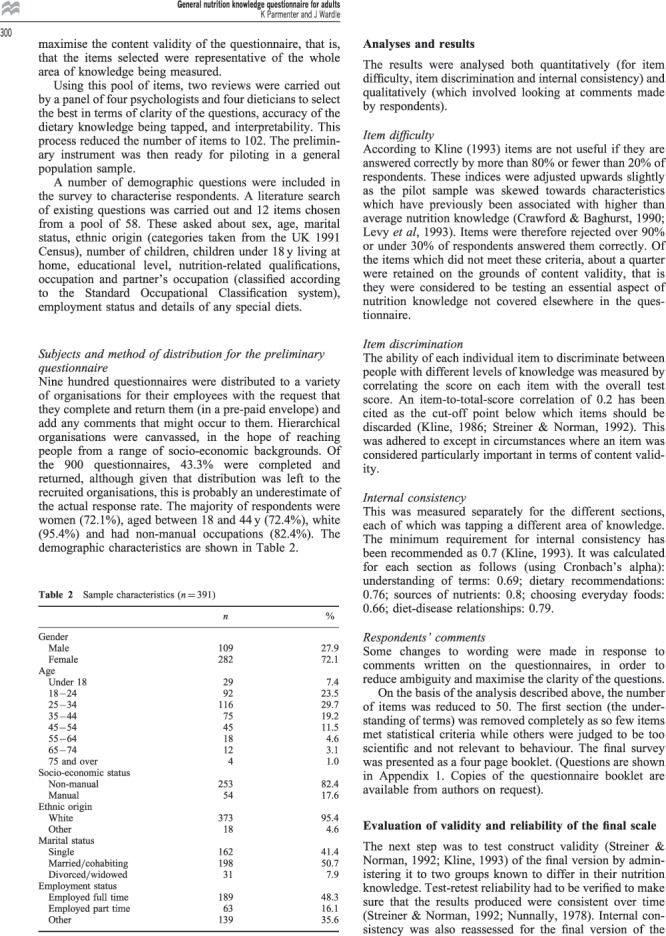
(Sheet 3).

**Supplementary File Q1:**
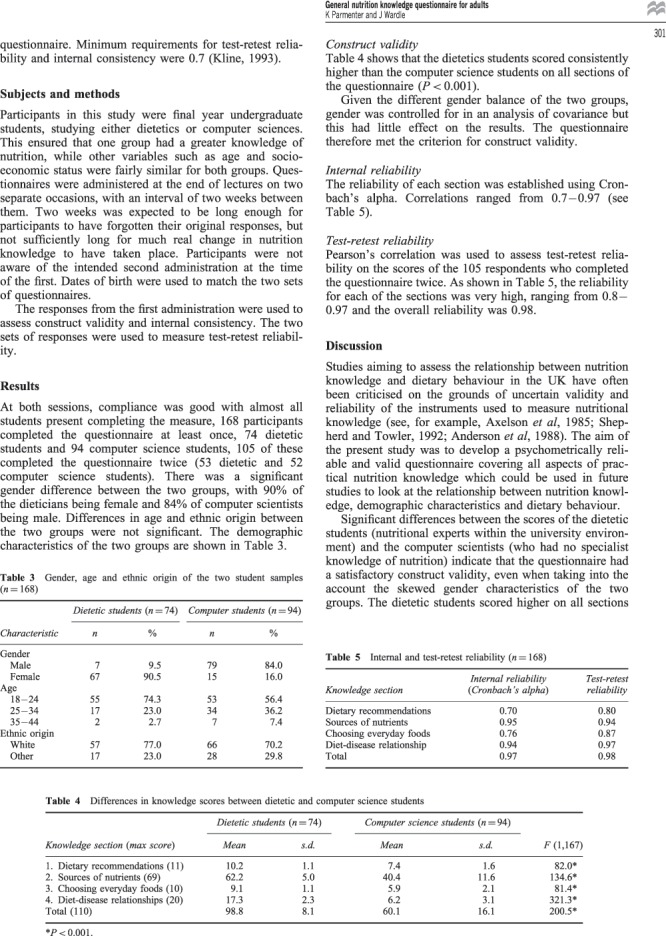
(Sheet 4).

**Supplementary File Q1:**
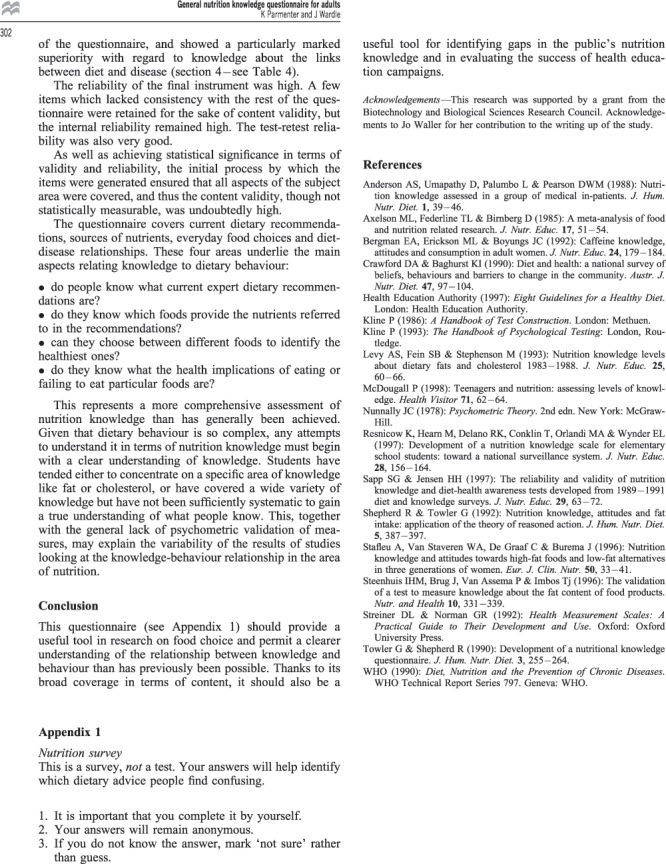
(Sheet 5).

**Supplementary File Q1:**
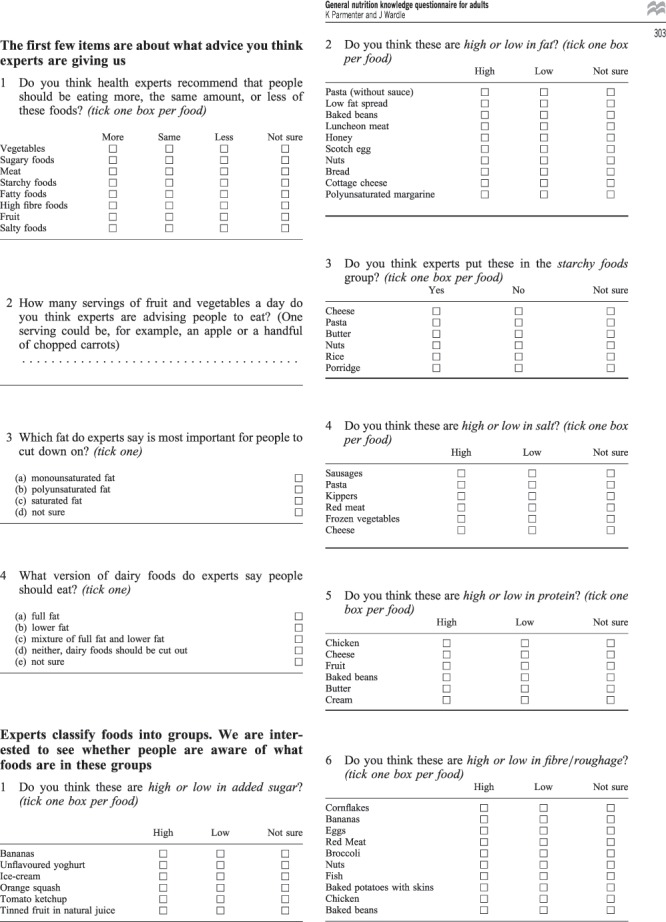
(Sheet 6).

**Supplementary File Q1:**
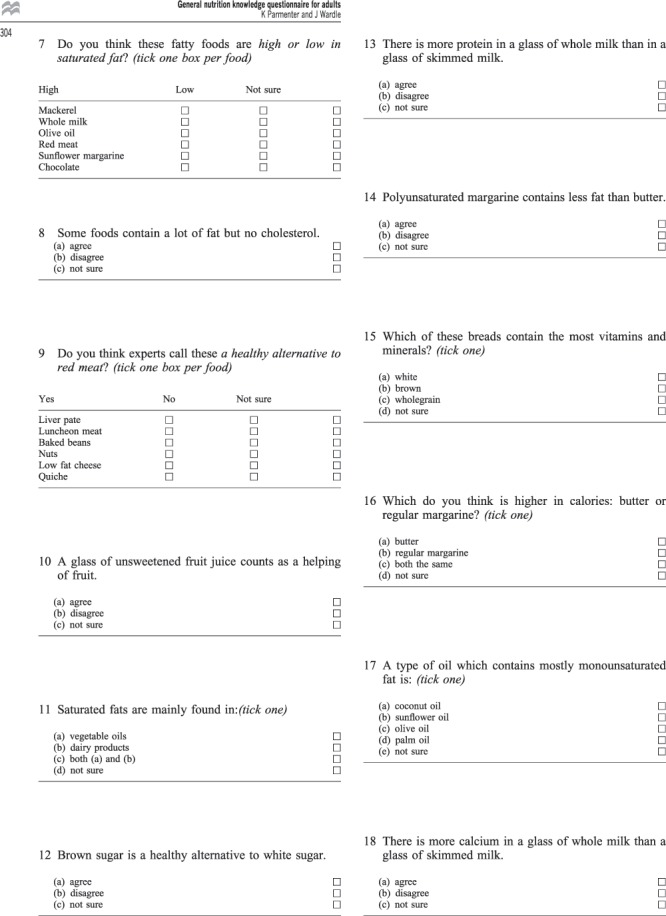
(Sheet 7).

**Supplementary File Q1:**
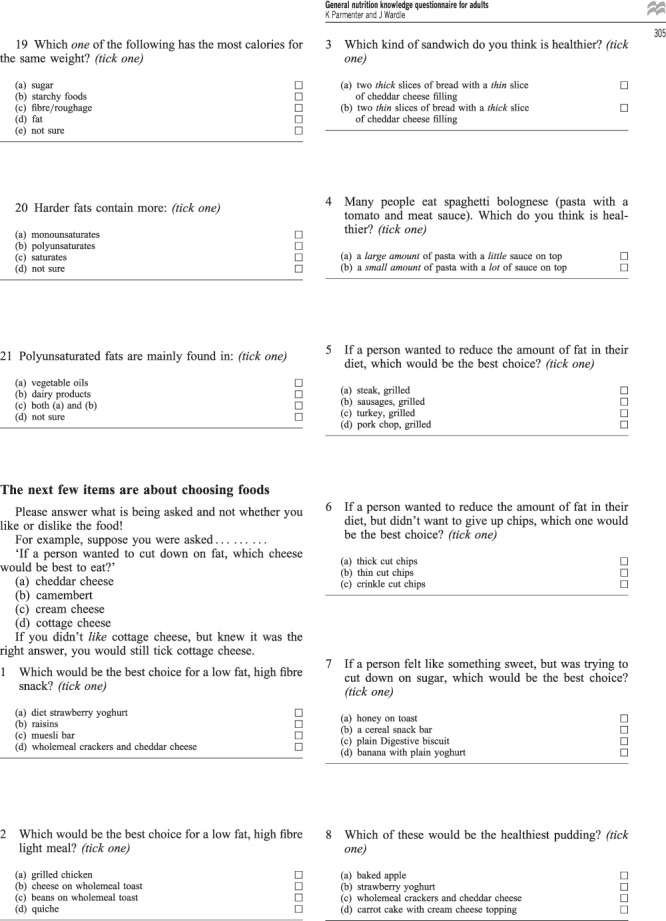
(Sheet 8).

**Supplementary File Q1:**
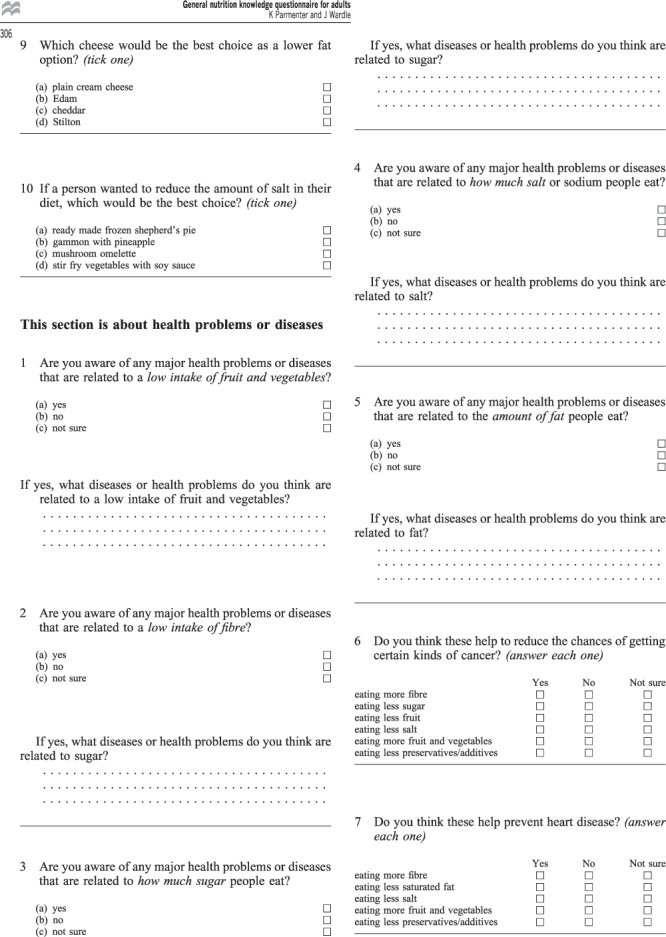
(Sheet 9).

**Supplementary File Q1:**
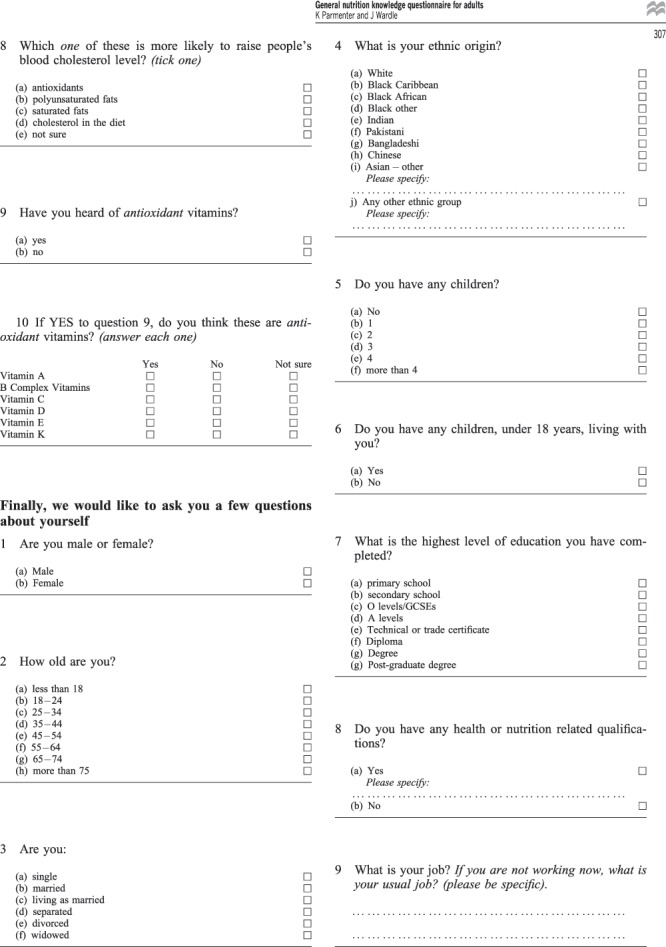
(Sheet 10).

**Supplementary File Q1:**
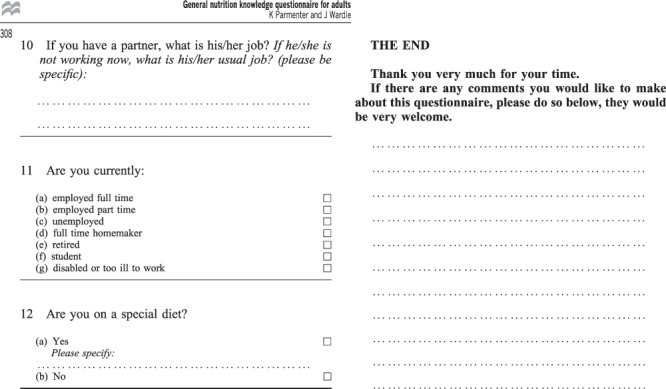
(Sheet 11).

**Supplementary File Q2:**
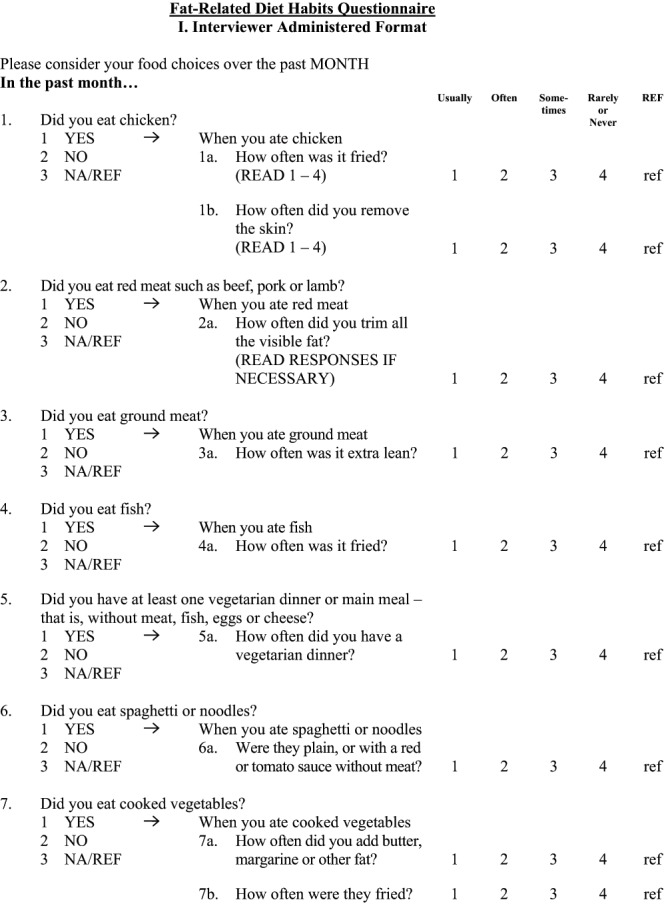
(Sheet 1).

**Supplementary File Q2:**
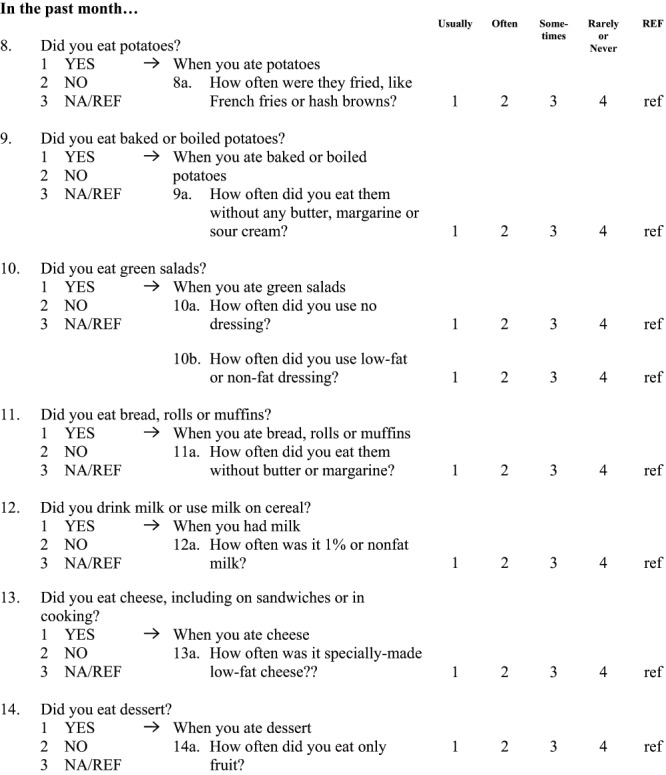
(Sheet 2).

**Supplementary File Q2:**
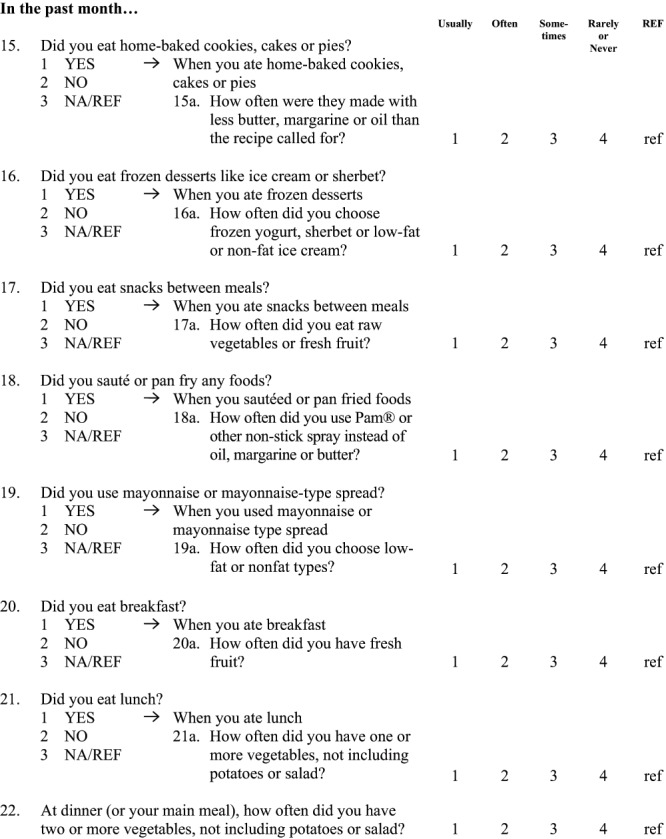
(Sheet 3).

**Supplementary File Q2:**
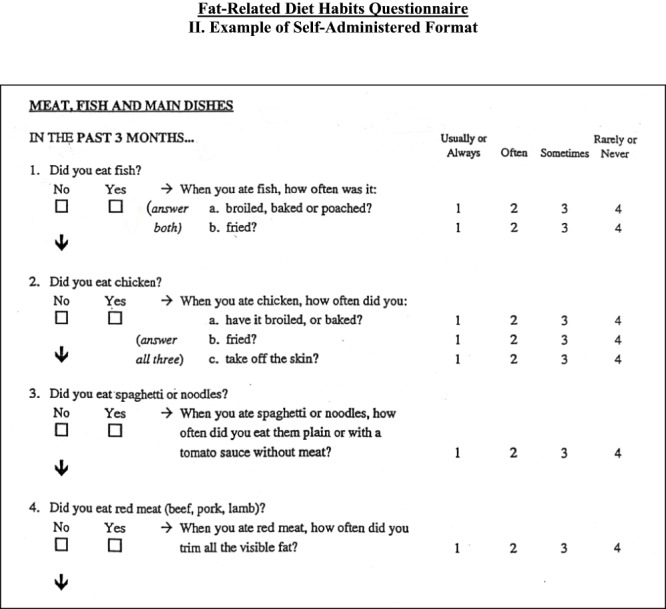
(Sheet 4).

**Supplementary File Q2:**
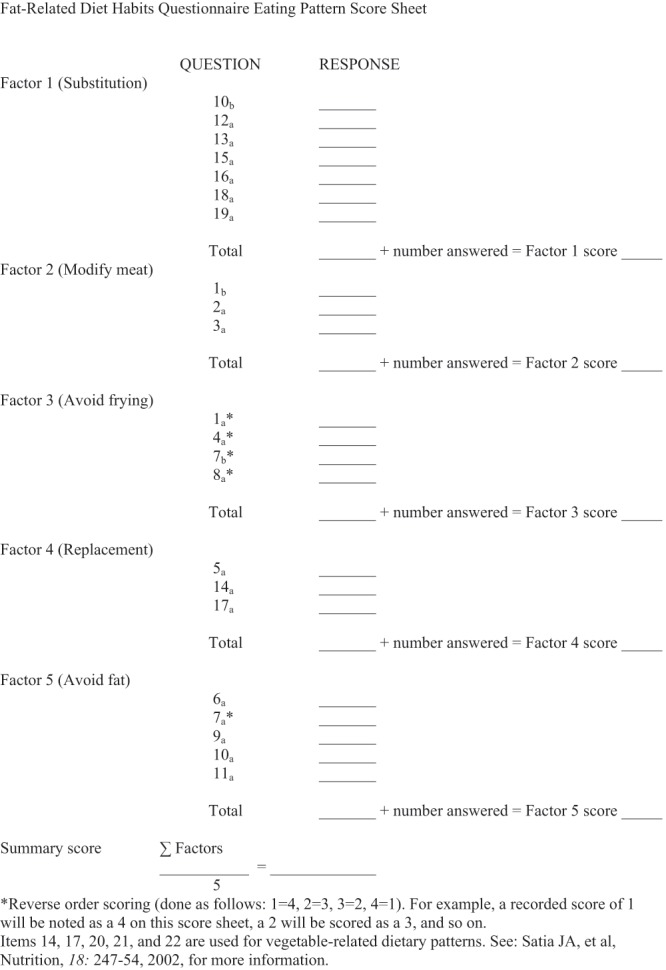
(Sheet 5).

**Supplementary File Q2:**
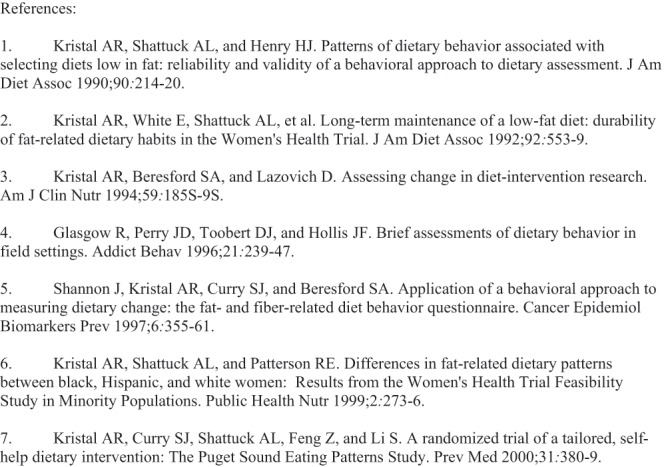
(Sheet 6).
