# Supplementary material for: Implementation of a worksite educational program focused on promoting healthy eating habits
Source: F1000Res. 2014 Mar 7;2:201. Originally published 2013 Oct 3. [Version 2] doi: 10.12688/f1000research.2-201.v2 (PMC4197742; doi:10.12688/f1000research.2-201.v2)
Supplement: Peer review history

# Peer review: Referee response for version 2

**DOI:** 10.5256/f1000research.3993.r5020

**Published:** 2014-06-06

**Authors:** Marjorie Freedman

**Affiliations:** 1 Nutrition, Food Science, and Packaging, San José State University, San José, CA, USA

I have read this submission. I believe that I have an appropriate level of expertise to confirm that it is of an acceptable scientific standard.

# Peer review: Referee response for version 2

**DOI:** 10.5256/f1000research.3993.r4049

**Published:** 2014-04-22

**Authors:** John Mooney

**Affiliations:** 1 School of Health and Related Research, University of Sheffield, Sheffield, UK

I confirm that satisfactory revisions have been made, thanks.

I have read this submission. I believe that I have an appropriate level of expertise to confirm that it is of an acceptable scientific standard.

# Peer review: Referee response for version 1

**DOI:** 10.5256/f1000research.2497.r3399

**Published:** 2014-02-18

**Authors:** Marjorie Freedman, Ashlee Gossard

**Affiliations:** 1 Nutrition, Food Science, and Packaging, San José State University, San José, CA, USA; 2 San José State University, San José, CA, USA

Thank you for the opportunity to review this paper. I have recently traveled to Greece and love traditional Greek food. It was distressing to read about low numbers of Greeks following a traditional Mediterranean diet, which has many health benefits. The authors are to be commended for undertaking this study in a worksite setting, aimed to lower fat intake. Overall, this research project represents a simple, non-invasive, low-cost method of attempting to improve health behaviors among a work force. It is unfortunate that the lack of evidence of significant change in actual health behavior is also typical of many such small-scale, short-term projects. The authors attempted to link change in knowledge with change in behavior. However, the literature is clear that change in knowledge alone is not sufficient to change behavior. The use of the Health Belief Model was presumably chosen to enhance motivation to change behaviors (even though over 60% of the population was at a normal weight, despite a “medium to large consumption of fatty foods.”). However, there was limited information presented in this paper as to how the constructs of the HBM were integrated into the classes presented to the employees. Did participants perceive any risks? Did they perceive susceptibility to a particular disease or to weight gain?  This needs further explanation. 

 Additional issues that need to be addressed are as follows:

1. The Supplemental files (Q1 and Q2) are presented inversely from their description in text.  The text indicates that the Knowledge questionnaire is File Q1, when it is in fact Q2, and the reverse is true for the Diet Habits questionnaire (actually Q1, listed as Q2 in text).  [minor technicality]
2.  Q1, Diet Habits, indicates that some portions of this questionnaire were administered by an interviewer, while other portions were self-administered by participants.  However, in text no mention is made of interviewer participation in the questionnaire, indicating only that participants received explanation in the Phase I session how to complete the form; greater clarification of procedure is required here. Furthermore, social pressures and positive presentation bias during personal interviews with a study administrator could have resulted in participants misrepresenting (toward a "healthy" direction) the quantities and frequencies of the foods they consumed. Particularly if the pre-intervention diet habit assessment was done via personal interview while the post-intervention was done via self-completed questionnaire, changes in dietary habits post-intervention may not have been accurately captured. This could also possibly explain the apparent increase in consumption of fried foods post-intervention.
3.  The diet habits questionnaire also asks participants about their food intake over the past month (~30 days) or 3 months; however, barely 30 days elapsed between the first day of intervention (Phase 2 - day 16) and the post-intervention questionnaire (Phase 4, day 45).  If dietary changes are to be captured, either a shorter-term assessment (i.e. asking about intake over the past 2 weeks) or a greater delay between intervention and post-assessment should be considered.
4.  The authors note that some participants took vacations during the intervention/assessment period.  Given the typical deviations from "normal" dietary habits that many people enjoy on vacation, and the recency (within the past month) of the diet habits questionnaire, this factor may function as a greater confounding variable in the observed lack of change in diet habits than the authors acknowledge.
5.  The 45-minute primary intervention lecture (Phase 2) was based on the Health Belief model, and it is the authors' claim that the HBM (which links beliefs and perceptions about health behaviors/risks to an individual's personal level of risk and his/her ability to control that risk through healthier behaviors) is an effective way to approach behavior change. However, Section 4 of the Knowledge questionnaire, which addresses the Diet-Health relationship, showed the least significant change following the intervention. Therefore, either the intervention lectures did not adequately target this subsection of knowledge, or else the participants were resistant to learning in this area for other reasons. The Discussion section, addressing the lack of observed behavior change, would be strengthened by acknowledging that the supposedly most critical piece of effecting behavior change was apparently not adequately targeted by the intervention, as evidenced by the lack of change in knowledge in this area.  In addition, 61% of participants were normal weight. Thus, it is unclear why the authors chose the Health Belief Model, and how the intervention was tailored to address perceived risk, perceived susceptibility, perceived barriers or perceived benefits. 
6. [another technicality/clarification] Table 4 (comparison of dietary habits) would be improved with a caption or footnote clarifying that a *lower* score on this assessment indicates *improved/more healthy* eating habits.

We have read this submission. We believe that we have an appropriate level of expertise to confirm that it is of an acceptable scientific standard, however we have significant reservations, as outlined above.

# Peer review: Referee response for version 1

**DOI:** 10.5256/f1000research.2497.r1969

**Published:** 2013-10-23

**Authors:** John Mooney

**Affiliations:** 1 School of Health and Related Research, University of Sheffield, Sheffield, UK

Many thanks for the opportunity to review this paper. Tanagra and colleagues present a concise, interesting and well discussed piece of research on their attempts to improve dietary practices in the workplace, using a ‘health belief model’ approach to improving awareness in the first instance of the main components of a healthy diet and food items which it is advisable to consume more sparingly (e.g. fried food). I particularly liked the focus for the most part on blue collar occupational groups and the relative homogeneity of the target population. This holds promise for being able to target higher risk occupational groups that are known to be less receptive to health promotion type interventions.

It is relatively well established among advocates of public health interventions, however, that changing actual behaviour is a much greater challenge than improving awareness of risk factors, particularly around diet and eating practices. While the contrast between the improved knowledge and no real benefit in behaviour is certainly of interest in this well controlled setting, it is not a terribly surprising or novel finding in its own right. As the authors themselves also point out, this is a study population which already has a relatively healthy diet, so their scope for encouraging and achieving improvements is necessarily limited. The narrow sample characteristics of the sub-group participating in the post-intervention follow-up (90% women) also restricts any meaningful inferences around the influence of gender and occupational group (a fact also acknowledged by the authors).

My most significant misgiving about the article in its present form however surrounds the established limitations of the ‘health belief model’ as applied to preventative public health interventions:

“ *The HBM is more descriptive than explanatory, and does not suggest a strategy for changing health-related actions. In preventive health behaviors, early studies showed that perceived susceptibility, benefits, and barriers were consistently associated with the desired health behavior; perceived severity was less often associated with the desired health behavior. The individual constructs are useful, depending on the health outcome of interest, but for the most effective use of the model it should be integrated with other models that account for the environmental context and suggest strategies for change.”*

[Extract from Boston University MPH content around ‘Limitations of the Health Belief Model’; http://sph.bu.edu/otlt/MPH-Modules/SB/SB721-Models/SB721-Models2.html].

The call for looking at the environmental context in particular suggests a potential missed opportunity within the current study to examine the characteristics and quality of food catering within the workplace itself.

Overall, as stated at the outset, I think this is an elegant and well-presented piece of research which certainly deserves to be published, but ideally needs some additional discussion around some of its methodological drawbacks. While the authors have acknowledged the short duration and sample size issues, there could be more discussion of desirability bias in self-reporting and ‘healthy-worker’ effects, the latter of which almost certainly will have diminished their scope for demonstrating improvements. Those relatively minor issues aside, there does need to be a fuller consideration of the limitations of the health belief model and the slightly confusing absence of any details on the types of catering and foods available within the workplace itself. In terms of specific essential revisions, I would suggest:

1. Demographic characteristics (Table 2): the addition of a section within 'individual characteristics' on how this population compares with the general Greek population on BMI and smoking etc.
2. Acknowledgement of the drawbacks of HBM and some description of the catering opportunities within the workplace itself, and if there are aspects of availability which could be improved to compliment the educational measures.
3. Some additional discussion around the drawbacks of self reporting and to what extent other back-up measures, such as 24 hour recall diaries, had been considered to enhance and substantiate the quality of the data gathered.

I have read this submission. I believe that I have an appropriate level of expertise to confirm that it is of an acceptable scientific standard.
